# Fluorescent Labeling of Polysaccharides from Masson Pine Pollen and Its Effect on RAW264.7 Macrophages

**DOI:** 10.3390/polym10040372

**Published:** 2018-03-26

**Authors:** Mengmeng Sun, Fangchen Su, Jinxin Yang, Zheng Gao, Yue Geng

**Affiliations:** 1 Shandong Provincial Key Laboratory of Animal Resistance Biology, School of Life Science, Shandong Normal University, Jinan 250014, China; sun007_2007@163.com (M.S.); sfc19900314@163.com (F.S.); yjx910702@163.com (J.Y.); 15266807498@163.com (Z.G.); 2 College of Bioengineering, Qilu University of Technology (Shandong Academy of Sciences), Jinan 250353, China

**Keywords:** polysaccharides, PPM60-Tyr-FITC, RAW264.7 macrophages

## Abstract

In order to explore the immediate effect of polysaccharides and macrophages, polysaccharides from masson pine pollen (PPM60) were labeled with fluorescein isothiocyanate (FITC) by using a chemical-derived method, and the reactant was named PPM60-Tyr-FITC. Direct interaction of PPM60-Tyr-FITC and RAW264.7 macrophages could be detected by flow cytometer (FCM), and this interaction could be inhibited by Pitstop 2 (clathrin inhibitor) and TAK-242 (Toll-like receptor 4 inhibitor). The results of confocal laser scanning microscopy (CLSM) also revealed that there was a co-localization phenomenon between PPM60-Tyr-FITC and RAW264.7 macrophage receptors, and it could be suppressed by Pitstop 2 and TAK-242. It was confirmed that PPM60 enters into RAW264.7 macrophages mainly through endocytosis, rather than the phagocytosis, and TLR4 played a mediating role.

## 1. Introduction

Though there are many research studies on polysaccharides from masson pine pollen, we know little about their intracellular location, metabolism, and identity, especially whether they exhibit effects extracellularly or intracellularly. Fluorescence probe technology has been widely used in chemical or biological research and analysis, because of its high sensitivity and selectivity [1,2]. Common fluorescent dyes include fluorescein, rhodamine, cyanide dye, and so on.

Polysaccharides have functions in immunoregulation, and can act as anti-virus, anti-tumor, and anti-aging agents. Cell receptors are important sites of polysaccharide function. A polysaccharide fraction from leaves of *Diospyros kaki* Thumb (PLE0) could activate RAW264. 7 and promote the secretion of NO, but anti-toll-like receptor 2 (TLR2) antibody reduced PLE0-induced NO production [3]. Polysaccharides from *Ganoderma atrum* (PSG-1) could increase the expression level of the mannose receptor (MR). When macrophages were pretreated with mannan (a MR inhibitor) before PSG-1, macrophage phagocytosis was inhibited obviously [4]. PS-F2 is an extracellular heteropolysaccharide fraction purified from the submerged mycelial culture of *G. formosanum.* Through the dectin-1 and complement receptor 3 (CR3), PS-F2 could identify macrophages and induce a series of signal changes [5]. Nakamura found that fucoidan induced the production of NO, then activated downstream signaling pathways, such as p38 MAPK and NF-κB, by the macrophage scavenger receptor (MSR) [6].

Endocytosis has emerged as critical pathway in regulating cell growth and resisting extracellular adverse factors. According to the state of the endocytic material, endocytosis is divided into phagocytosis and pinocytosis. Depending on whether it relieson receptors, endocytosis is further divided into receptor-mediated and nonspecific endocytosis [7]. Pinocytosis can occur in almost all eukaryotic cells, but phagocytosis often presents in specialized cells, such as macrophages. Pathways of endocytosis mainly include phagocytosis, clathrin-dependent pathways [8], clathrin-independent pathways [9], such as lacuna-dependent pathways [10], GEEC (glycosyl phosphatidylinositol-anchored protein enriched endosomal compartments) pathways [11], macropinocytosis [12], and so on.

FITC-dextran could enter cells by receptor-mediated endocytosis [13] and nonreceptor- mediated endocytosis [14]. Many plant polysaccharides, such as *Ganoderma lucidum* polysaccharides, could also enter cells [15] or promote the endocytosis of lipopolysaccharides (LPS) [16]. In recent years, a series of research studies also suggested that TLR4 involved in endocytosis. Endocytosis was activated by the interaction between cell surface ligands and Toll-like receptor 4 (TLR4), and induced MyD88-independent TRAM signal pathway [17], while the early stage of endocytosis relied on clathrin.

Our lab has previously conducted many studies of PPM60. These confirmed that PPM60 plays an important role in the adjustment of immunoregulation and in regulating immune cells through TLR4 signal pathway [18,19]. However, whether polysaccharides from masson pine pollen could be absorbed into the cell was unclear. Besides, there were many research studies showing that TLR4 was the receptor of the polysaccharides [20,21,22]. In addition, due to fact that the molecular weight of FITC-dextran is close to masson pine pollen polysaccharide, PPM60, and could enter into cells by receptor-mediated endocytosis, we selected FITC-dextran as a positive control for research in our study.

## 2. Materials

RAW264.7 macrophages (Chinese Academy of Sciences, Shanghai, China). Fluorescein isothiocyanate-dextran (FITC-dextran), chloroquine phosphate (CQ, Sigma-Aldrich, Saint Louis, MO, USA). TAK-242 (Calbiochem, Darmstadt, Germany). Hoechst 33342, bovine V, cytochalasinB (CB, solarbio, Beijing, China). Pitstop 2 (Abcam, Bristol, UK). Cy3-Goat anti-rabbit IgG (BosterBio, Wuhan, China).

Carbon dioxide cell incubator (Nuaire, Plymouth, MN, USA). StatFax-2100 BIV-TEK INSTRUMENTS INC. (Awareness, Portsmouth, VA, USA). Inverted microscope (Olympus, Tokyo, Japan). Confocal laser scanning microscope (Leica, Solms, Germany). Flow Cytometer (Becton Dickinson, East Rutherford, NJ, USA). Ultimate 3000 High performance liquid chromatograph (Thermo, Waltham, MA, USA) with a refractive index detector.

## 3. Methods

### 3.1. Purification, Detection, and Fluorescent Labeling of PPM60

#### 3.1.1. Preparation of Polysaccharides

Polysaccharides were extracted using the method of water-boiling and ethanol deposition from wall-broken pollen of *Pinus massoniana*. Sixty percent ethanol-deposited polysaccharides, named PPM60, with better activity were used in this experiment. Then, 200 mg PPM60 was labeled with Tyr by tyramine reduction method. PPM60 (200 mg) was dissolved in 7.5 mL 0.2 mol/L phosphate buffer (pH 8.0), followed by 200 mg tyramine and 75 mg sodium borohydride in that order, and reacted for 96 h at 37 °C. After reaction, the separation was conducted by centrifugation, and the collected liquid freeze-dried; the reactant was named PPM60-Tyr [14,21].

#### 3.1.2. Purification of PPM60-Tyr-FITC

The reducing end of PPM60-Tyr could undergo nucleophilic reaction with FITC. PPM60-Tyr (200 mg) was weighed and dissolved in water, adjusted pH to 8.5 using 0.5 mol/L NaHCO_3_, and added 25 mg FITC at room temperature, and reacted overnight. Then, the reactant was added to ethanol at 80% final concentration. There was a large amount of bright yellowish- green precipitate, and centrifugation was needed to collect the precipitate. The precipitate was dissolved into water and precipitated by ethanol three times; the reactant was named PPM60-Tyr-FITC. The later was purified through gel filtration chromatography using Sephacryl S-400 HR gel. The whole process conducted was above while avoiding light exposure [15,23].

#### 3.1.3. Determination of Fluorescent Substitution of PPM60-Tyr-FITC

To determine the marked efficiency of PPM60 with FITC, a regression equation was made. FITC solution was prepared by adding 1 μg/mL FITC solution to 0.25, 0.5, 0.75, 1.0, and 1.25 mL in five test tubes, and adding distilled water to 2.5 mL; the whole process should avoid light. Fluorescence intensity of each tube was measured using fluorescence spectrophotometer (Ex = 488 nm; Em = 530 nm), with distilled water set as a blank control. The standard curve was drawn, with FITC concentration as the horizontal coordinate, while fluorescence intensity Abs was the ordinate. Sample solution was made by adding 1 mg PPM60-Tyr-FITC to 5 mL distilled water, then, 0.5 mL sample solution mixed with 1.5 mL distilled water. The fluorescence intensity of the latter was measured and the substitution degree was calculated with the regression equation [15].

### 3.2. Effect of PPM60-Tyr-FITC on RAW264.7 Cell Viability

Cell plating concentration was 2 × 10^4^/mL, and cells was seeded in 96-well plates at 200 μL per well. The control group, PPM60, and PPM60-Tyr group were set at 200 μg/mL, PPM60-Tyr-FITC group at 200, 400, 800, 1600 μg/mL, respectively, and each concentration was with 8 replicates.

### 3.3. Influences of PPM60-Tyr-FITC on RAW264.7 Macrophages

TAK-242, cytochalasin B, and Pitstop2 were dissolved by DMSO; the final concentration was 10 µg/mL. CQ and NH_4_Cl solution were prepared with PBS, and their final concentrations were 20 and 500 µg/mL, respectively.

#### 3.3.1. Observation of the Fluorescence Changes by CLSM

##### Cell Climbing Experiment

Macrophage suspensions were adjusted to 2 × 10^4^/mL. The proper cell suspension was incubated in a poly-lysine-treated culture plate with.

TAK-242 and Pitstop 2 groups: after cell climbing, TAK-242 or Pitstop 2 at the final concentration of 10 μg/mL was added and cultured for 30 min. Then, 400 μg/mL FITC-dextran or PPM60-Tyr-FITC was added and cultured for 20 min. Primary antibody was added after fixation, and then secondary antibody; the nucleus was stained with Hoechst 33342 for 5 min and sealed by glycerol. In other groups, the process is the same as above. Their concentrations and times are as follows: CB group, 10 μg/mL, 20 min; NH_4_Cl group, 10 μg/mL, 30 min; CQ group, 20 μg/mL, 30 min.

##### Observation of Each Group with CLSM

Prepared slides could be observed immediately under CLSM or stored at −20 °C in a refrigerator.

##### Analysis by Flow Cytometer

When observed by CLSM, the inhibitory effect of CB, CQ, NH_4_Cl on co-localization was not obvious, so we did not choose them in the follow-up experiments.

After culturing for 24 h, macrophages were treated with FITC-dextran or PPM60-Tyr-FITC dissolved by DMEM medium for 20 min. Macrophages were collected using a cell scraper, and washed using PBS. Each group was counted and adjusted to 2 × 10^6^ cells/mL, and 0.5 mL cell suspension was used for flow analysis. In TAK-242 and Pitstop 2 groups, the inhibitors were added before fluorescently labeled polysaccharides.

## 4. Results

### 4.1. Fluorescent Labeling of Polysaccharides from Masson Pine Pollen

#### 4.1.1. Tyramine Labeling of Polysaccharides

**Figure 1:**
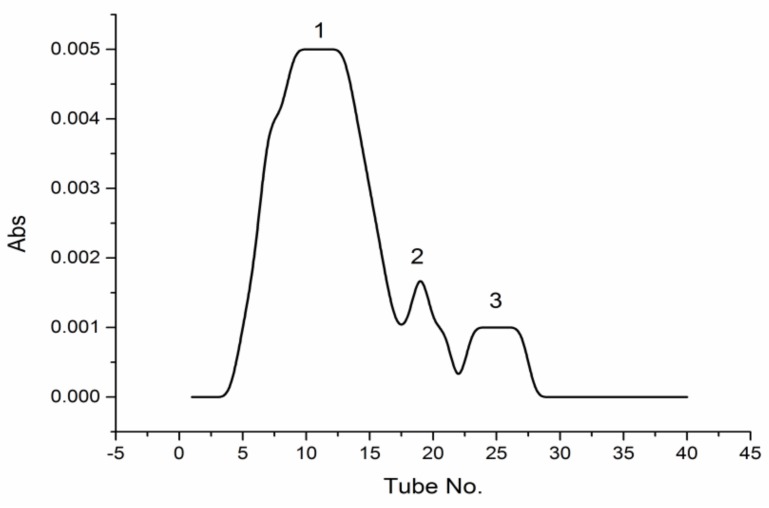
The Sephacryl S-400HR chromatogram of PPM60-Tyr.

Labeled PPM60-Tyr (10 mg) was dissolved in distilled water and purified through gel filtration chromatography using Sephacryl S-400HR (GE Healthcare, Uppsala, Sweden), and eluent collected every 5 min, with optical density measured at 280 nm. The results showed that PPM60-Tyr had three absorption peaks. The concentration of the first peak was highest between 4–17 tubes (Figure 1).

**Figure 2:**
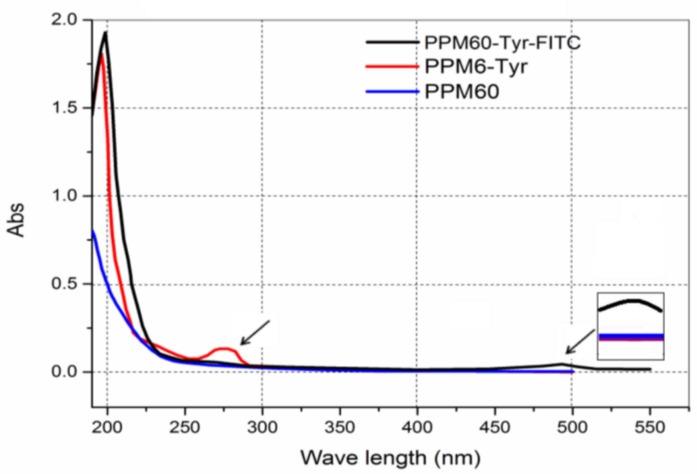
Spectral scanning curves.

Peak 1 was merged and scanned at 190–500 nm. Only one more peak at 280 nm (Figure 2, red line) was shown compared to PPM60, indicating that Tyr was bound to the PPM60.

#### 4.1.2. The Results of FITC Labeling

Product labeled with FITC was named PPM60-Tyr-FITC. By UV scanning, there was a new absorption peak at 490 nm (Figure 2), indicating that FITC was bound to the PPM60.

The freeze-dried PPM60-Tyr-FITC was dissolved with distilled water and purified with Sephacryl S-400HR. OD value was measured at 490 nm to test the FITC. The concentration of polysaccharide was determined by phenol-sulfuric acid method [24] at 490 nm. Fluorescence absorption was measured by fluorospectrophotometer. Four peaks were obtained and named peak 1, peak 2, peak 3 and peak 4.

**Figure 3:**
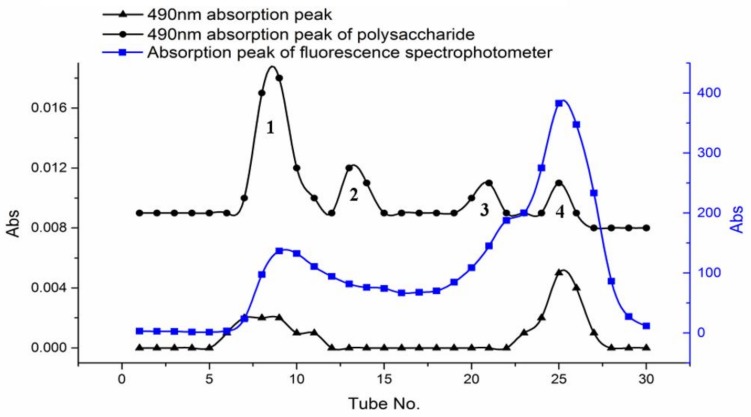
The results of all the absorption peaks.

In Figure 3, we could see two overlapping peaks at peak 1 and 4 of polysaccharides. It suggested that peak 1 and peak 4 were all labeled with both FITC and Tyr. The purified constituent of peaks 1 and 4 were lyophilized, and yielded 75.2 and 163.5 mg, respectively.

#### 4.1.3. Determination of Substitution Degree and Molecular Weight of PPM60-Tyr-FITC

**Figure 4:**
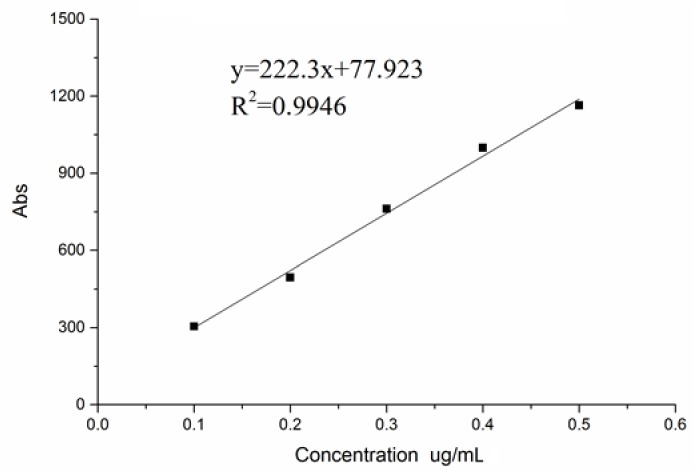
The standard curve of FITC.

The regression equation can be seen in Figure 4.

The fluorescence substitution degree of peak 1 was 4.32%. Compared with the 0.66% of substitution degree of peak 4, we chose the peak 1 for the follow-up experiment, due toits high substitution degree.

The average molecular weight was measured by gel chromatography. According to the standard curve, logMw = −0.4283*t* + 12.738 (*R*^2^ = 0.9789), the molecular weight of peak 1 was Mw = 316 K. It is close to 500 K Mw of FITC-dextran. Therefore, we adopted FITC-dextran as a positive control.

### 4.2. Effects of PPM60, PPM60-Tyr, and PPM60-Tyr-FITC on RAW264.7 Macrophages

**Figure 5:**
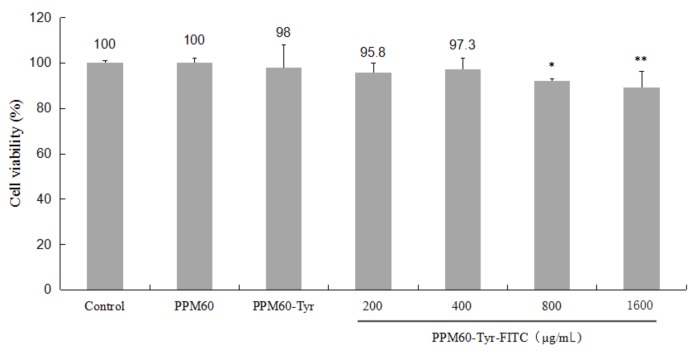
The influences of PPM60, PPM60-Tyr, PPM60-Tyr-FITC on the activity of RAW264.7 macrophages. The control group, PPM60, and PPM60-Tyr group were set at 200 μg/mL, PPM60-Tyr-FITC group at 200, 400, 800, 1600 μg/mL, with each concentration composed of 8 replicates. ** *p* < 0.01 vs. Control, * *p* < 0.05 vs. Control.

Compared with the control, PPM60-Tyr had a slight effect on RAW264.7 macrophages. High concentrations of PPM60-Tyr-FITC (800, 1600 μg/mL) showed significant inhibiting effects (Figure 5). In the subsequent experiment, we used 400 μg/mL as the working concentration.

### 4.3. Immunofluorescence Research on the Changes of Fluorescence after FITC-Dextran Entersthe RAW264.7 Macrophages

**Figure 6:**
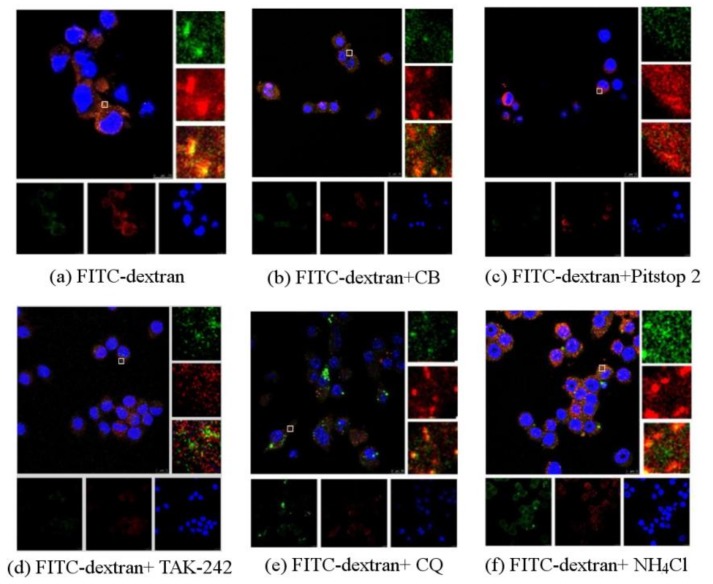
Observation the influences of FITC-dextran on RAW264.7 macrophages by CLSM (scale bar = 10 μm) (**a**) Green fluorescein was FITC, red was antibody, yellow was the co-localizationof polysaccharides and antibodies, blue was nucleus. In the absence of any inhibitor, there was co-localization, presented in yellow; (**b**) the group of cytochalasin B also had co-localization phenomenon. Pitstop 2; (**c**) or TAK-242; (**d**) group had no co-localization; CQ (**e**) or NH_4_Cl (**f**) had little effect on RAW264.7 macrophages.

Without any inhibitor, there were bright yellow spots, representing the co-localization of PPM60 and receptor (Figure 6a). The group with cytochalasin B also had a co-localization phenomenon (Figure 6b), indicating that CB had little effect on the co-localization of PPM60 with its receptor. Pitstop 2 (Figure 6c) group and TAK-242 group (Figure 6d) had no co-localization, illustrating that they could inhibit the binding of fluorescent PPM60 to cell surface receptors. After addition of CQ (Figure 6e) and NH_4_Cl (Figure 6f), there was little effect on RAW264.7 macrophages.

### 4.4. FCM Detection of the Effect of Pitstop 2 and TAK-242 on FITC-Dextran Entering the RAW264.7 Macrophagesin Various Time Periods

**Figure 7:**
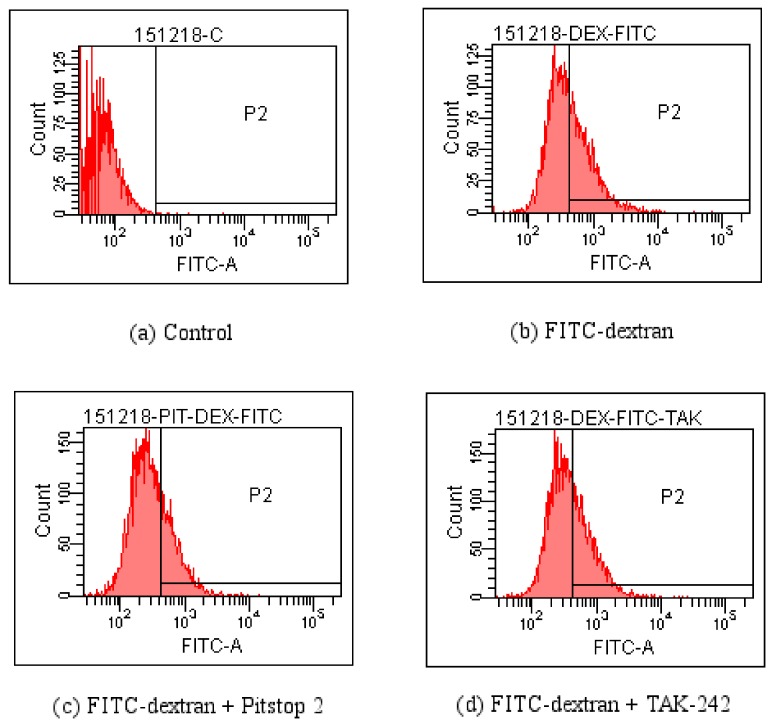
Detection influences of Pitstop 2 and TAK-242 on the entrance of PPM60-Tyr-FITC into RAW264.7 macrophages by FCM. (**a**) Control; (**b**) FITC-dextran; (**c**) FITC-dextran + Pitstop 2; (**d**) FITC-dextran + TAK-242

The results of flow cytometer further demonstrated that the entering of FITC-dextran polysaccharide was related to the pinocytosis. The results were shown in Table 1 and Figure 7.

### 4.5. The Changes of Fluorescence after PPM60-Tyr-FITC Entersinto RAW264.7 Macrophages

**Figure 8:**
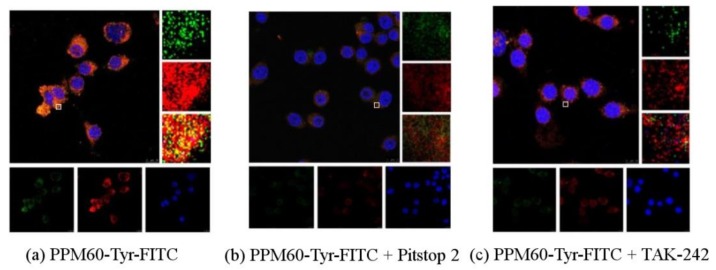
Observation of the influences of PPM60-Tyr-FITC on RAW264.7 macrophages by CLSM (scale bar = 10 μm). (**a**) Co-localization of PPM60-Tyr-FITC and receptor was observed without inhibitor; (**b**) Pitstop 2 group had no co-localization; (**c**) TAK-242 group had no co-localization.

**Figure 9:**
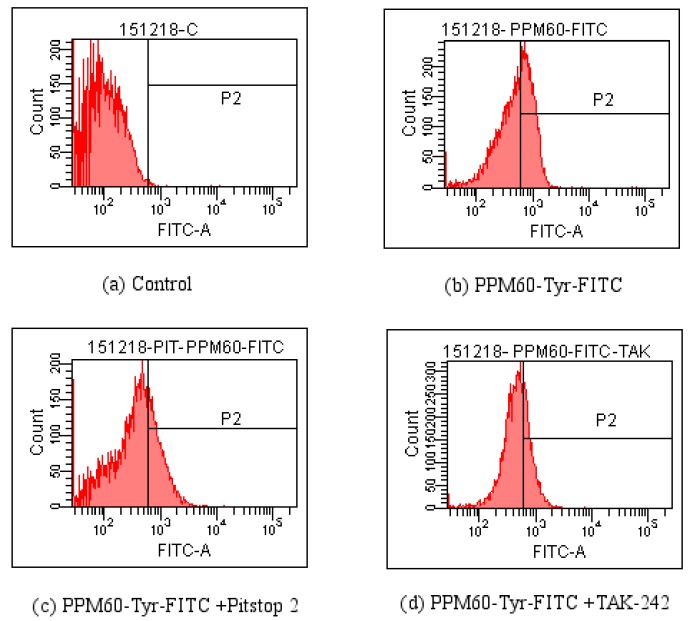
Detection influences of Pitstop 2 and TAK-242 on the entrance of PPM60-Tyr-FITC into RAW264.7 macrophages by FCM. (**a**) Control; (**b**) PPM60-Tyr-FITC; (**c**) PPM60-Tyr-FITC + Pitstop 2; (**d**) PPM60-Tyr-FITC + TAK-242.

As seen from Figure 8, co-localization of PPM60-Tyr-FITC was observed without inhibitor (Figure 8a). Pitstop 2 (Figure 8b) and TAK-242 group (Figure 8c) had no co-localization, which showed that both of them could inhibit the binding of fluorescent PPM60 to cell surface receptor. This result corresponded to the results of flow cytometer, shown in Table 2 and Figure 9.

## 5. Discussion

In this study, the polysaccharide from masson pine pollen was successfully labeled with FITC through tyramine reduction method, which laid a basic foundation and provided the experimental basis for future research of polysaccharide. At the same time, it also provides a basis for the study of the role of polysaccharide in cells and the signaling pathway. There has been much research on the interaction between polysaccharides and receptors. Some studies have suggested that polysaccharides interacted with receptors on the cell surface, such as TLR4 [25], scavenger receptor [6], dectin-1 [26], and so on, then triggered the changes of signaling pathway (MAPK, NF-κB) and second messenger (Ca^2+^). Some reporters believed polysaccharides cross the cell membrane and play a role in the cell by means of endocytosis. Some research has even shown that both of them are involved in the modulation between the polysaccharides and TLR4 by the molecules of CD family, such as CD-14 [27], and this process was involved in the change of signal and endocytosis.

This set of experiments was on the basis of predecessors’ research, and through this study, we further confirmed the mediating role of TLR4 receptor, and endocytosis was a way for masson pine pollen polysaccharide to enter into cells.

The CLSM results showed the co-localization of FITC-dextran and TLR4, which appeared brightly yellow (Figure 6a) because of co-localization between FITC-dextran marked with green fluorescence and TLR4 marked with red fluorescence, that indicated that dextran interacted with TLR4. The fluorescence of yellow in co-localization declined after treating with TAK-242 (Figure 6d), which indicated that TLR4 was indeed the receptor for polysaccharides. The yellow fluorescence was also significantly attenuated by the addition of the inhibitor Pitstop 2 (Figure 6c), indicating that clathrin-mediated endocytosis was involved in the process, as also stated before. The co-localization did not change much after adding CB compared with the control group (Figure 6b), which meant there was little FITC-dextran component entering into cells through phagocytosis, which needs the contraction of actin microfilaments. We did not see any obvious differences after adding CQ and NH_4_Cl, which influences the degradation of components of intracellular lysosomes by regulating proton pumps (Figure 6e,f), suggesting that the inhibition of endosomal acidification did not directly accelerate the disappearance of co-localization between FITC-dextran and Cy3-tagged TLR4.

As there was little inhibitory effect after treating with CQ and NH_4_Cl, and less influence of CB, these inhibitors were not used in experiments on PPM60-Tyr-FITC and RAW264.7 macrophages. TAK-242 and Pitstop 2 could inhibit the effect of FITC-dextran on cells, and the inhibition rates were 36.4% and 18.6% (Table 1) when PPM60-Tyr-FITC were 31.4% and 40%, respectively (Table 2). The co-localization of PPM60-Tyr-FITC and TLR4 was also detected by CLSM, but disappeared after adding TAK-242 and Pitstop 2. The results confirmed that masson pine pollen polysaccharide, which has a molecular weight of 316 K, mainly entered RAW264.7 macrophages through endocytosis, rather than phagocytosis, with TLR4 playing a mediating role.

The results showed that after interaction of LPS and TLR4, both entered the endosome to form a complex structure [28]. There are two signal pathways correlated with LPS and TLR4. One induces the TIRAP-MyD88 signaling pathway, and the other induces a MyD88-independent TRAM-TRIF signaling pathway [29] which relies on the Syk/PLCγ2-mediated endocytosis. Could masson pine pollen polysaccharide be involved in this way? Could it be digested after going into the intracellular endocytosis body? Could it react with intracellular receptor? These questions deserve further study. Neither clathrin inhibitor nor TLR4 inhibitor in our experiment could fully suppress the polysaccharide entering into cell, which suggests polysaccharides are being absorbed and then reacting in the cell or may be involved in other pathways. In addition, monomeric G protein Rabs such as Rab7b [30] and Rab10 [31], also regulate TLR4 signal pathway. Therefore, further experiments should be performed in terms of regulating proteins.

## 6. Conclusions

This study labeled polysaccharides from masson pine pollen PPM60 with FITC successfully, and the reactant was named PPM60-Tyr-FITC. Direct interaction of PPM60-Tyr-FITC and RAW264.7 macrophages could be detected by FCM and CLSM, and this interaction could be inhibited by clathrin inhibitor Pitstop 2 and TLR4 inhibitor TAK-242.It was confirmed that PPM60, with a molecular weight of 316 K, entered into RAW264.7 macrophages mainly through endocytosis, rather than phagocytosis, and that TLR4 played a mediating role.

## Figures and Tables

**Table 1:** Percent of cells having FITC signal among all the RAW264.7 macrophages.

Group	Percent (%)
Control	0.3
FITC-dextran	46.1
FITC-dextran + Pitstop 2	37.5
FITC-dextran + TAK-242	29.3

**Table 2:** Percent of cells having FITC signal among all the RAW264.7 macrophages.

Group	Percent (%)
Control	0.4
PPM60-Tyr-FITC	46.2
PPM60-Tyr-FITC + Pitstop 2	30.5
PPM60-Tyr-FITC + TAK-242	31.7
